# Patient Experiences following Acute HIV Infection Diagnosis and Counseling in South Africa

**DOI:** 10.1371/journal.pone.0105459

**Published:** 2014-08-25

**Authors:** Benjamin J. Wolpaw, Catherine Mathews, Yolisa Mtshizana, Mickey Chopra, Diana Hardie, Mark N. Lurie, Virginia De Azevedo, Karen Jennings

**Affiliations:** 1 South African Medical Research Council, Health Systems Research Unit, Cape Town, South Africa; 2 University of Cape Town, School of Public Health and Family Medicine, Cape Town, South Africa; 3 University of Cape Town, Department of Virology, Cape Town, South Africa; 4 Brown University Medical School, Department of Community Health, Providence, Rhode Island, United States of America; 5 Cape Town Municipality Health Directorate, Cape Town, South Africa; Alberta Provincial Laboratory for Public Health/University of Alberta, Canada

## Abstract

Individuals in the acute stage of HIV infection (AHI) have an elevated potential to transmit HIV and play a critical role in the growth of the epidemic. Routine identification and counseling of individuals during AHI could decrease transmission behavior during this key period. However, diagnosis of AHI may present challenges distinct from those experienced through diagnosis of established HIV infection. A study was conducted in a public youth clinic outside of Cape Town, South Africa, to identify and counsel individuals with acute stage HIV infection. In-depth interviews were conducted with patients following diagnosis. After counseling, patients were accepting of the testing regimen used to diagnose AHI. They used the knowledge of having been recently infected to identify the source of their infection, but did not retain or place importance on information regarding the increased ability to transmit HIV during the acute stage. Future interventions directed at the reduction of HIV transmission following diagnosis with AHI will need to find ways of making this information more salient, possibly through more culturally meaningful educational approaches.

## Introduction

Individuals in the acute stage of HIV infection (AHI) have an elevated potential to transmit HIV and play a critical role in the growth of the epidemic. The acute phase lasts for the first weeks to months following infection, during which time high viral burden leads to greatly increased infectiousness, while rapid HIV antibody tests yield negative or indeterminate results [1], [2], [3]. As a result, patients who present for testing during the acute stage often remain unaware of their HIV infection, even as they experience a temporary period of elevated infectiousness [1], [4], [5]. Diagnosis of AHI requires the use of more expensive, laboratory based nucleic acid amplification testing (NAAT) or HIV antigen testing [1].

Routine identification and counseling of individuals during acute HIV infection could decrease transmission behavior during this key epidemiological period, but presents unique challenges. Since acutely infected individuals will test negative on a rapid antibody test and then positive on NAAT, there is the potential for confusion or mistrust of the seemingly contradictory results. Those who do become aware of their HIV infection during the acute phase will know that they were recently infected and that they are in a period of temporarily elevated infectiousness. How this information might be understood and interpreted is unclear. Furthermore, it is particularly concerning in the context of the known potential for HIV positive status disclosure to provoke violent reactions towards women, particularly well described in the antenatal settings [6].

The one other group to perform qualitative research on this topic in Southern Africa found that a majority of individuals continued engaging in sexual activity after being diagnosed with AHI, and that understanding of increased infectiousness during AHI was limited [7]. Furthermore, use of a motivational interviewing-based counseling intervention among this population did not reduce sexual risk behavior compared to educational sessions alone [8]. Other research has revealed significant confusion over testing procedures and a poor understanding of the health and transmission consequences of AHI in a population consisting mainly of men who have sex with men in the US [9]. Thus, further characterization is needed of patients following diagnosis with AHI in order to better understand the barriers to effective education and counseling.

Here we present findings from in-depth interviews that were conducted with those diagnosed during AHI at a public youth clinic outside Cape Town, South Africa. This study adds to the limited body of research investigating the unique challenges of AHI diagnosis in resource poor areas.

## Methods

The study was conducted in the Khayelitsha township of the Western Cape of South Africa, in a public youth clinic offering STI treatment, voluntary counseling and testing (VCT), and family planning services to patients under 26 years old. Khayelitsha has high unemployment and many people live in abject poverty – 25% of households report having no income and over 50% of houses are informal dwellings or shacks [10], [11].

**Figure 1:**
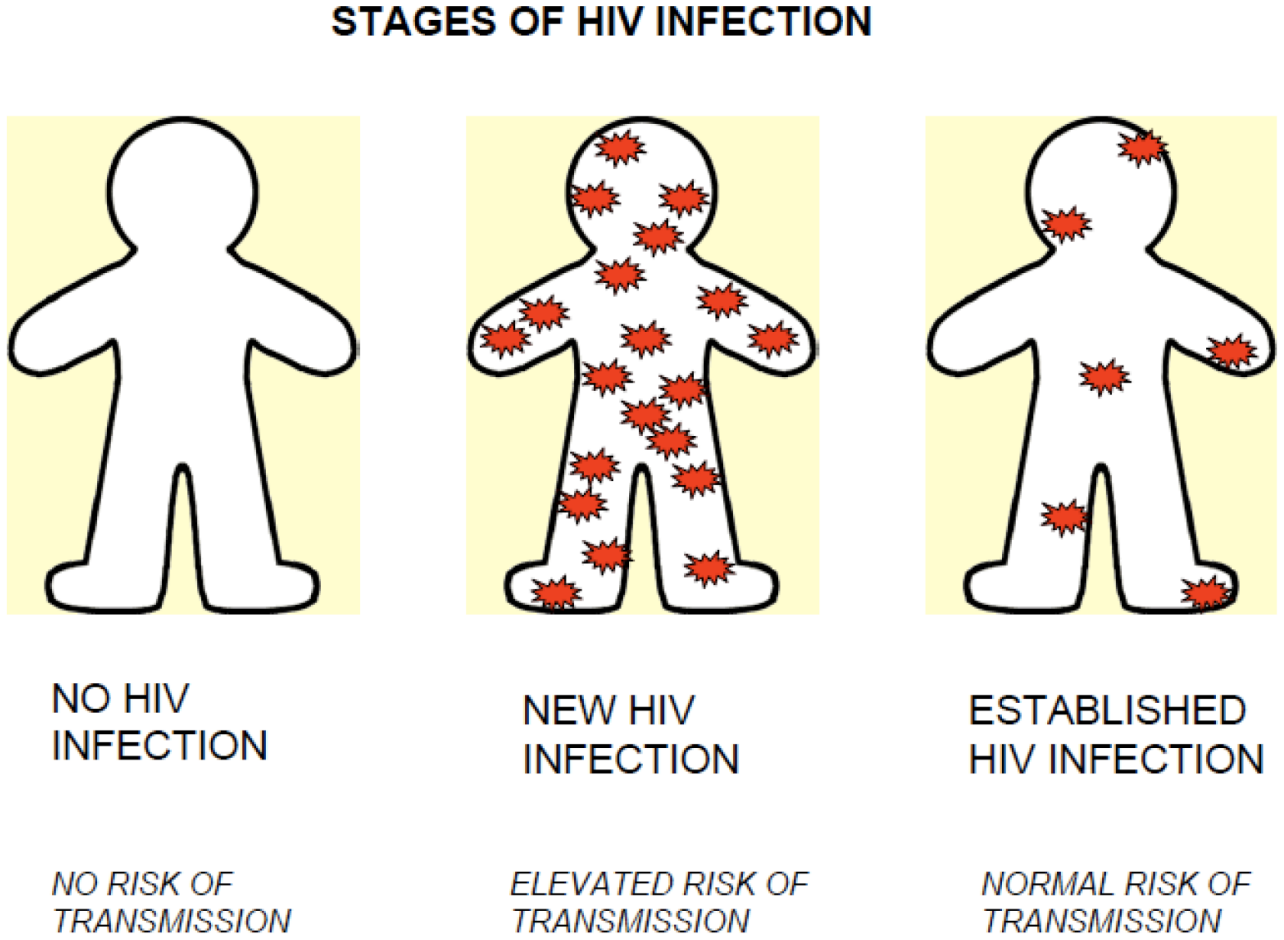
AHI Transmission Diagram.

The details of how patients were enrolled and tested and a description of the study population have been reported elsewhere [12]. Briefly, those VCT and STI patients 18 and over who tested negative for HIV at the clinic were referred to an on site study location for NAAT testing. Informed consent, data collection, and all counseling were conducted in the patient's native language (isiXhosa). Written documentation of informed consent was obtained. Standard HIV pre- and post-test counseling for acutely infected participants was supplemented with teaching about AHI including the fact that acutely infected particpants would have originally received a negative result as well as discussion of the transient high levels of viremia in acute infection that could increase the probability of transmitting HIV. The goals of counseling were to help participants process and understand the test results, provide psychosocial support, and to encourage them to take steps to avoid transmission of HIV. Figure 1 shows a tool that was used to explain transiently elevated transmission potential during AHI.

Acutely infected participants were asked to return for an interview one week following the receipt of positive results. This interview was performed in the participant's own language (isiXhosa) by a researcher experienced in qualitative research. An interview guide approach was utilized where the interviewer decided the sequence and wording of questions based a predetermined outline [13]. The topics covered were: events since receiving results, feelings about self, feelings about and interactions with partners, and understanding of topics covered during counseling.

Two researchers performed the analysis of interview findings through several steps. First, each researcher read the data independently for content and to identify patterns. The researchers communicated to settle on a coding scheme, based on the most important themes from the data. Relevant portions of the interview transcripts were coded, and then sorted according to theme. The coding sorts were further reduced into the most relevant information from which interpretations were hypothesized. These interpretations were then verified by returning to the coding sorts and original transcripts.

This research study received approval both the Human Research Ethics Committee of the University of Cape Town and the Brown University Institutional Review Board. It was planned and conducted in consultation with the Department of Health for the city of Cape Town.

## Results

Over the course of one year, 902 (80.0%) of 1128 eligible participants enrolled in the study. Six participants were identified with AHI; all were diagnosed, counseled, and interviewed. Table 1, copied from our previous publication in the Journal of Sexually Transmitted Infections, provides the codes by which each participant will be referred to in this paper along with descriptive information [12].

**Table 1:** Characteristics of AHI Cases.

	VCT or STI Patient	Gender	Age	Years of Education	Used Condom at Last Sex	Believe Partners Have Other Partners	Total Sexual Partners in Past Two Months
5987	VCT	Male	20	9	yes	no	3
8783	VCT	Female	24	11	no	unsure	1
8106	STI	Male	22	10	no	yes	2
2535	STI	Female	22	11	no	no	1
5036	STI	Female	19	9	no	no	2
8529	VCT	Female	19	8	no	no	1

Each of these individuals spoke of a significant emotional response such as shock, anger, or depression upon learning of their positive HIV status. They all mentioned fears about dying of AIDS and one mentioned contemplating suicide (5987). Although some had reached a level of comfort with their positive HIV status by the time of the interview, others were still significantly distressed.

The testing regimen appeared to have been fairly well accepted by participants, but there were points of confusion. Patient 8783 did not believe the results and chose to repeat rapid testing at another location. This patient did not initially mention concern over the testing regimen as the reason for wanting to retest. However, when asked if this might have contributed to her lack of confidence in the initial testing results, the patient agreed that “yes even that [was a factor] because really at the beginning they said it is negative.”

Patient 2535 opted to bring her partner in for testing without disclosing to her partner that she had tested or been diagnosed as positive. It was agreed that she would therefore be administered a rapid HIV test along with her partner, as if she had not yet been tested. Although the counselor explained beforehand that the patient might again test negative on the rapid test, the patient was confused when this did indeed occur:

Interviewer: That test maybe… how did you feel when you received negative results and his were positive?

Participant: I felt all right, but I did not understand what we were doing there.

Interviewer: You did not understand?

Participant: No

Interviewer: What exactly did you not understand?

Participant: That I had negative results and [my boyfriend] had positive results.

Initially, the patient remembered being told that this might happen but could not explain why it had occurred. Upon reminder by the interviewer of the different sensitivities of the two tests to early infection, the patient accepted this explanation of the discrepancy.

Beyond the details of the testing regimen, interviewers tried to determine whether the participants understood what acute HIV infection was, and the implications of this stage of infection. Participants had absorbed the fact that they were infected recently. They did not, however, seem to remember anything about temporarily increased infectiousness. The following example from participant 8106 demonstrates the typical extent of comprehension of AHI (diagram referred to in figure 1):

Interviewer: What is it that [the counselor] said?

Participant: She only told me that you working about it only, like people who are newly infected only.

Interviewer: There is nothing that you can think of maybe?

Participant: I can't remember a thing my sister.

Interviewer: Do you still remember her showing this diagram (figure 1) to you

Participant: No, I can still remember

Interviewer: Oh okay what do you remember that she told you about this diagram and what did you understand

Participant: No she said she said here, if it's new its like this it's all over your body. But here maybe it's when you do right things you see maybe you eat healthy things maybe it becomes like this.

Interviewer: Try to remember, is there anything that you can recall again, like what it means when the virus is all over.

Participant: she said, she said…

Interviewer: What did she say?

Participant: Yo I can't remember a thing I don't want to lie.

Interviewer: You can't remember like about the importance of using a condom here.

Participant: it's what I am telling you I can't remember a thing

Like this patient, others also mainly interpreted the stages of infection as an example of how they could best take care of themselves – you are more sick at the beginning but then can become healthier through a positive lifestyle. The most consistent messages taken away from the counseling session seemed to have to do with eating healthy foods. When asked what else they should do, participants did not know saying, “some of the things that I must not eat, I think that you talked about that and that I should exercise…I do not remember the other ones” (8783) or “doing exercises…having a healthy diet…I do not know anymore” (5987). Some mentioned condom use as being important to prevent infecting others or to prevent reinfection. Increased infectiousness during the acute stage did not enter into these considerations.

The knowledge of having been recently infected was clearly used by participants to indentify the source of their HIV infections. The female participants reported fewer partners prior to diagnosis and were fairly sure of who had infected them. The male participants had had more partners prior to diagnosis, but used the recency of infection to narrow down the number of suspected individuals. Participant 8106 explained, “when that lady told me that I was newly infected I said to her she must take this [partner on list] on top out because I was with her during September last year. So she does not feature in this HIV thing at all or it does not affect her and she can't be blamed.” Participant 5987 was also attempting to determine the source of infection, saying “I found myself HIV positive and that happened after I had met [one particular partner] because it is during those months that I contracted the virus. The results prove that my infection is recent.” The response to the knowledge about who may have been the source of infection varied from acceptance to anger, but when asked how the patient's felt about these individuals, there was no indication of any potentially violent response.

Participants did not have well formed plans as to how they would moderate the risk of infecting others. Three participants (8783, 5036, 2535) had disclosed their positive HIV status to their partners. However, overall participants were still focused on themselves at the time of interview, and had spent less time considering the implications of their infection for others. Participant 2535 explained her main concern, “what kept crossing my mind was the question of having a baby when I wanted one…that is what I was thinking about.” There was a belief among some that current partners would already be positive and thus it was not necessary to use condoms with that person. This reasoning was used even by one of the males (8106) who had multiple partners and did not know the source of his infection: “I told myself that we are both infected and I didn't see any need for us to use condom.” Patient 5036 believed her partner was positive, but planned to use condoms in order to prevent reinfection. As she explained “I told [my boyfriend] that if we don't use the condom we are making it to spread inside.”

When discussing the possibility of new partners, participants said that they were planning to use condoms to prevent passing on the virus. Patient 5036 stated “at the moment I am planning to use condoms all the time.” Another patient had apparently not regained her sex drive, saying “even if I were to meet with someone new, I don't have [sex] on my mind” (8783).

## Discussion

These interview findings shed light on how the unique aspects of acute HIV infection diagnosis and counseling will be understood and dealt with by patients. Participants in our study were generally accepting of the testing regimen, trusting the results of the laboratory based tests even though they contradicted the clinic rapid test. This was following counseling, however, and there was evidence to suggest that this area does offer the potential for confusion on the part of patients. Clear explanation of the tests used must be emphasized when testing for AHI is performed.

We had little success getting participants to understand and place value on the nature of acute infection including transiently elevated transmission potential. This information was explained to participants in plain, non-scientific language several times and a diagram (figure 1) was used as a visual aid. Still, during the interview participants did not demonstrate any retention of this information beyond recollection when heavily prompted by the interviewer. If the concept of elevated infectiousness is intended to be used in post-test counseling sessions to motivate patients to practice safe sexual practices, work must be done to develop a sound strategy for explaining and emphasizing this key point.

The participants included in this study come from backgrounds of low socioeconomic status and limited education, which may influence understanding of the testing, diagnoses, and implications related to AHI. Of note, a similar lack of understanding of AHI infection was also observed in a study performed in the United States with a relatively more educated patient population and range of incomes [9].

Participants placed significant importance on healthy eating and exercise as a way to take care of themselves over the long term. It was clear that the participants were most concerned with their own health at the time of the interviews, which might explain why healthy eating emerged as such a salient point. There were other strategies, however, discussed during counseling as being important for ensuring the health of the people living with HIV such as regular doctor visits and CD4 counts so that anti-retroviral treatment could be provided when necessary. These points did not arise during interviews, possibly because healthy eating may fit in better with the traditional, non-biomedical beliefs about health and disease that are important to many in South Africa. Future strategies should try to find ways to better integrate key public health messages about AHI with the belief system of patients.

Participants clearly understood that they had been recently infected, and used this information to determine as best they could who had been the source of their infection. Although this did not result in any signs of imminent violent confrontation in the cases that we observed, this may emerge as an important issue if AHI diagnosis is implemented on a broader scale. Previous research has shown fear of a violent reaction is a barrier to HIV disclosure and up to 14.6% of pregnant women reported experiencing such a reaction from a partner following disclosure [6].

Although the commitment to condom use with current partners was inconsistent among participants, there was evidence of a desire to prevent transmission to new partners in the future. This suggests the potential for transmission behavior to be reduced following AHI diagnosis. However, the patient's primary focus on their own health and limited understanding of the implications of AHI for transmission are barriers to achieving such an effect. A more thorough behavioral counseling approach, applied across multiple visits, may be more effective in overcoming these barriers. Reductions in transmission behavior have been demonstrated in the context of routine HIV diagnosis as well as a study of AHI diagnosis in the United States [14].

Pettifor et al. similarly reported a limited reduction in high transmission risk behavior following diagnosis with AHI and a deficient understanding of increased infectiousness among patients in Malawi and South Africa [7]. This group also recently published a trial investigating a motivational interviewing based counseling intervention for new AHI diagnosis [8]. Our study represents the only other report on this topic in sub-Saharan Africa, and compared to the Pettifor study our population was significantly younger (mean age 21 years vs 25.6). The similarity of our results supports the generalizability of these previous findings to the youth context. Our study also adds to the previous literature by bringing to light the way in which the knowledge of recent infection can be used to identify the source of infection, the potential for confusion surrounding discordant rapid testing and laboratory testing results, and the gap between the biology of high transmission risk and the medical beliefs of patients in this setting.

This study is limited due to the small number of participants interviewed, which was a function of the relatively low prevalence of AHI among the 902 participants enrolled. A larger study based on the findings presented here would be required to fully evaluate the feasibility and effectiveness of an intervention focused around AHI diagnosis. The small sample size was also insufficient to investigate differences between men and women following AHI diagnosis. This study both encouraged safe sexual practices and then interviewed participants about them, creating the potential for social desirability bias to influence the responses of the participants during interviews with study staff. Rather than a source of hard conclusions, this data is intended to supplement the one previous study on AHI in sub-Saharan Africa, and to provide an evidence base to guide future intervention and research with the goal of routine diagnosis and behavioral counseling of patients during acute HIV infection.
